# Pain levels and typical symptoms of acute endodontic infections: a prospective, observational study

**DOI:** 10.1186/s12903-016-0222-z

**Published:** 2016-05-27

**Authors:** Dan-Krister Rechenberg, Ulrike Held, Jakob M. Burgstaller, Gabriel Bosch, Thomas Attin

**Affiliations:** Department of Preventive Dentistry, Periodontology and Cariology, Center of Dental Medicine, University of Zürich, Switzerland, Plattenstrasse 11, CH-8032 Zürich, Switzerland; Department of Internal Medicine, Horten Center for Patient Oriented Research and Knowledge Transfer, University of Zürich, Zürich, Switzerland

**Keywords:** Pain, Root canal, Symptom, Diagnostic

## Abstract

**Background:**

This study aimed to identify key symptoms that could be associated with the diagnosis of acute forms of symptomatic apical periodontitis (SAP) and symptomatic irreversible pulpitis (SIP), and to identify a diagnostic algorithm based on these symptoms.

**Methods:**

In this prospective, observational study 173 emergency patients diagnosed with acute pain of endodontic origin and no swelling or fistula were included. Patients were asked 11 specific questions from a checklist with a possible discerning value between acute SAP and acute SIP. Pain levels were recorded using the numeric rating scale (NRS-11). Subsequently, the painful tooth was diagnosed. Logistic regression was used to evaluate the checklist regarding its differentiation between SAP (*N* = 103) and SIP (*N* = 70). Moreover, a decision tree was constructed based on recursive partitioning to identify a hierarchy in differentiating symptoms.

**Results:**

With identical median NRS-11 scores of 8, the teeth diagnosed with acute SAP and SIP were severely painful. The decision tree analysis resulted in a tree with splits according to pain on cold, perceived tooth extrusion, and pain duration. The overall sensitivity of the tree to detect SAP based on key symptoms was 95 %, its specificity was 31 %.

**Conclusions:**

The best indicator for SAP was a reported absence of pain to cold stimuli. In teeth that did have a history of pain triggered by cold stimuli, the decision tree correctly identified SAP in 72 % of the teeth that felt too high and had hurt for less than one week.

## Background

Odontalgia is the main cause of oro-facial pain [1]. Acute dental pain is mostly attributed to microbial infection of the dentin being in close proximity to the pulp, the pulp space and finally the periapical tissues [2, 3]. The pulp, the periodontal ligament, and the periapical tissues form natural barriers, which help the host orchestrate a defense against invading opportunistic pathogens [4]. When these barriers are invaded with pathogens nociceptors are activated due to inflammation and tissue breakdown [5]. Three symptomatic clinical conditions deriving from endodontically involved teeth have been identified: symptomatic irreversible pulpitis (SIP), symptomatic apical periodontitis (SAP) and acute apical abscess (AAA) [6]. Even though these can be extremely painful [2, 7], they do not need to be [8, 9]. Unfortunately, the current diagnostic nomenclature of the American Association of Endodontists (AAE; [6]) does not differentiate between teeth that cause significant enough pain to require the patient to seek emergency care from those, which merely show an increased reaction to diagnostic tests [10].

The socioeconomic importance of dental pain has long been recognized [11]. It has thus been attempted to develop specific dental pain questionnaires for epidemiologic studies [12]. These questionnaires appear to have good predictive values to differentiate between groups of conditions, such as those that are caused by endodontic infections and those that are not. However, they do not differentiate the main causes of severe dental pain emanating from endodontic origin. There have been other approaches, which were more specific. However, these frequently mixed symptoms reported by the patients with clinical observations by the investigators [13, 14]. The significance of establishing the correct diagnosis of endodontic infections should not be under-estimated [15]. If endodontic infections are not treated appropriately, life-threatening conditions can evolve [16]. Due to the obvious clinical symptom of edema (swelling) associated with the diagnosis of AAA, this diagnosis poses no challenge. In contrast, clear-cut symptoms have not been identified to differentiate between SAP and SIP. While SIP is merely painful, SAP is the beginning of the spread of infection with a possibility of untoward systemic consequences [16]. Abscess formation, where bacteria are invariably present in the periodical tissues, marks one possible endpoint of this infection process [17]. Depending on the severity of the infection and the location of the tooth, SAP can even lead to the death of the patient if not treated appropriately [18]. Furthermore, the emergency treatment for SIP and SAP differs [15]. With SIP, simply removing the coronal pulp is sufficient for relief [19], while with SAP the disinfection of the entire root canal system is needed. It would thus be helpful to further investigate symptoms including pain levels and pain duration that can be specifically related to the acute forms of SIP and SAP.

In this prospective, observational study, adult patients seeking emergency care in a dental hospital because of inflammatory conditions caused by infection of the pulp space were assessed. The aim of the study was to identify key symptoms that could be associated with either SIP or SAP, and to identify a diagnostic algorithm based on these symptoms. Symptoms were related to clinical signs/findings.

## Methods

### Cohort identification and inclusion criteria

**Fig. 1:**
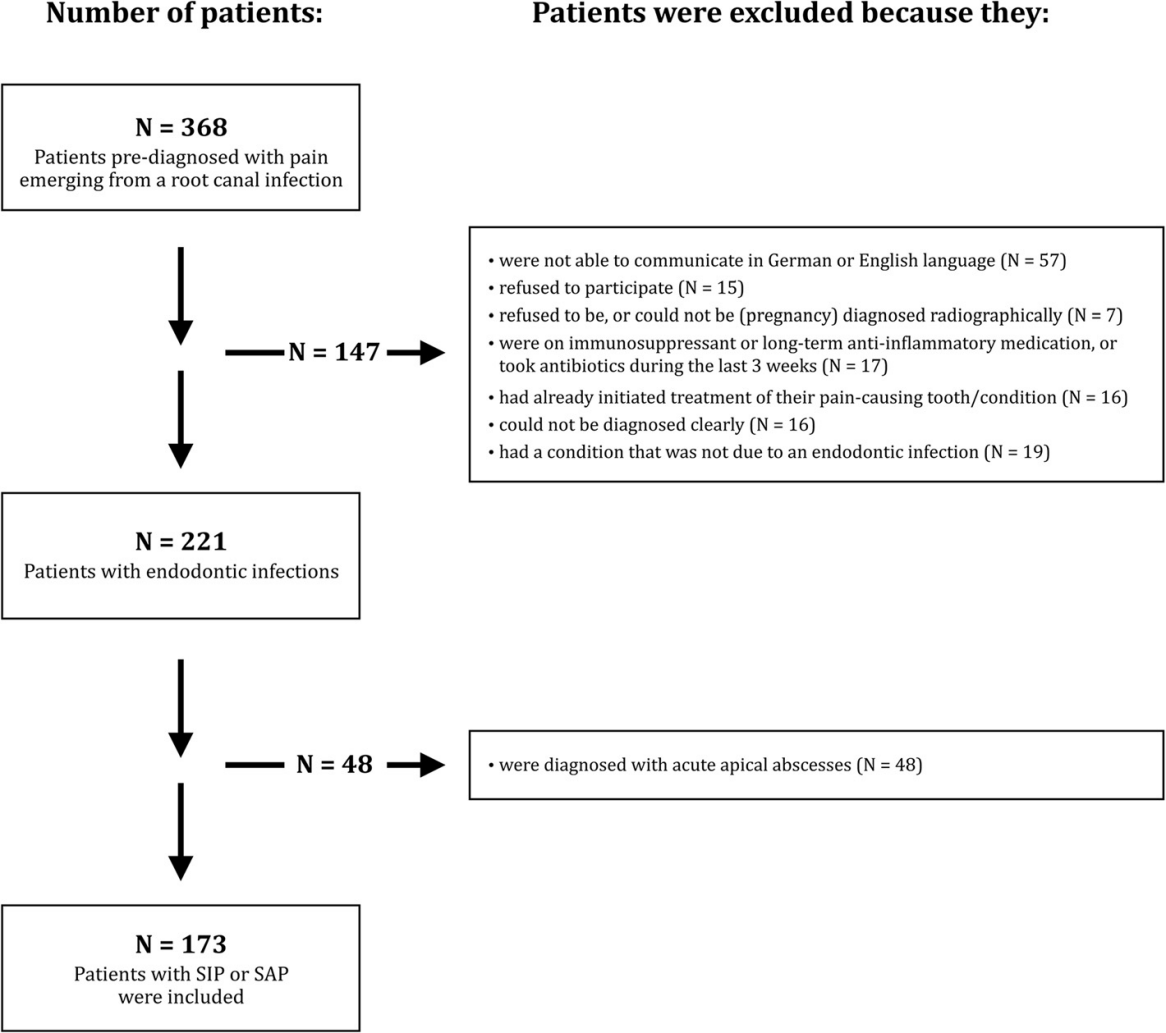
Flow chart depicting the decisions why patients were excluded from the study. All emergency patients presenting with severe pain originating from a single endodontically involved tooth in the course of 64 weeks were initially considered. Teeth diagnosed with an acute apical abscess were then excluded. *SIP* symptomatic irreversible pulpitis, *SAP* symptomatic apical periodontitis.

All patients attending the dental emergency unit at our institution from opening at 07:30 am to 10 am were considered. It was aimed to include all adult (18 years or older) patients presenting with acute pain from a permanent tooth caused by an endodontic infection. The emergency unit consisted of physicians and dentists from all dental specialties. After a short first examination by an oral surgeon, patients diagnosed with pericoronitis or temporomandibular joint pain were referred to the Oral Surgery department. All other patients were referred to the Department of Preventive Dentistry, Periodontology and Cariology for further examination. Patients who did not present with spontaneous pain, but merely reported slight discomfort to stimuli indicative of reversible pulpitis were not considered as’acute’. These patients did not enter the study. The remaining patients presenting with acute pain from a permanent tooth were asked to participate in the study (Fig. 1). Written informed consent was obtained from all patients. The current study protocol was approved by the local ethics committee (KEK-ZH-Nr. 2012-0450) and was conducted in accordance with the Declaration of the World Medical Association. Moreover, it was confirmed that this investigation conformed to STROBE guidelines for observational studies. Patients were excluded from the study if they: (i) were not able to clearly communicate in German or English language, (ii) refused to participate, (iii) refused to be, or could not be (pregnancy) diagnosed radiographically, (iv) were on immunosuppressant or long-term anti-inflammatory medication, or took antibiotics during the last 3 weeks, (v) had already initiated treatment of their pain-causing tooth/condition, (vi) could not be diagnosed clearly, (vii) had a condition that was not due to an endodontic infection, or (viii) were diagnosed with an acute apical abscess.

### Checklist for key symptoms and pain assessment

Participants who fulfilled the inclusion criteria were interviewed using a checklist with 11 dichotomous questions with a possible discerning value between SIP and SAP (Table 1). This checklist listed symptoms that have been reported in the endodontic literature [20]. It also contained an assessment of pain intensity and duration. The checklist was originally written in German and translated to English for international patients. It was piloted by the authors of this communication and later filled in by either one of two investigators (DKR and GB). Patients were guided through these questions by one of these two investigators. Patients who could not communicate clearly were excluded. In addition, the pain intensity of the presenting condition was assessed using the numeric rating scale (NRS-11; [21]). The examiner asked the patient to quantify his/her maximum pain intensity within the last 24 h on a scale from 0 to 10. The following anchors were used to describe the rating scale:’0’ = no pain/pain free and ‘10’ = worst pain imaginable.

**Table 1:** History checklist for dental emergency patients asking for symptoms to possibly discern between SIP and SAP

#	Question	Answer options
1	Has the pain interfered with sleep?	y/n
2	Has the pain ever been stronger than during the last 24 h?	y/n
3	Did the pain start less than 1 week ago?	y/n
4	Has the pain been sporadic or constant?	sporadic/constant
5	Has the pain been localized or radiating?	localized/radiating
6	How was the main quality of the pain?	dull/sharp
7	Has chewing increased the pain?	y/n
8	Have warm drinks/food increased the pain?	y/n
9	Has cold increased the pain?	y/n
10	Has cold decreased the pain?	y/n
11	Does the affected tooth feel too high?	y/n

*SIP* symptomatic irreversible pulpitis, *SAP* symptomatic apical periodontitis

### Diagnosis

The clinical evaluation included cold testing with carbon dioxide snow, assessment of tenderness to percussion, tooth mobility, and periodontal probing depths. Moreover, the soft tissues were checked for tenderness to palpation, signs of erythema, and presence of a sinus tract or swelling. The findings were compared to a healthy, contralateral tooth that served as a control. Radiographic examination was performed using single-tooth radiographs (Digora, Soredex, Tuusula, Finland). The different inflammatory endodontic conditions (SIP, SAP, or AAA) were established according to the recommended diagnostic terminology of the Consensus Conference of the AAE [6]. In a deviation from that nomenclature, however, each tooth was assigned only one main diagnosis (Table 2). The examiner noted this diagnosis, together with relevant data (date, gender, age, analgesics taken in the past 24 h) in an anonymized data sheet.

**Table 2:** Clinical findings used in the current study to differentiate between SIP and SAP

Criterion	SIP	SAP
Sensitivity to carbon dioxide snow	+	-
Radiographically widened ligament space	+/-	+
Periapical radiolucency	-	+/-
Swelling or sinus tract	-	-

*SIP* symptomatic irreversible pulpitis, *SAP* symptomatic apical periodontitis, + positive response or sign clearly present, +/- mixed response or not present in all cases, - negative response or clearly absent

Note: sensitivity to percussion was not included, as almost all of the acutely painful teeth in this study responded positive to percussion

### Statistics and data evaluation

Descriptive statistics included counts and percentages for the questions on pain history. Categorical data was compared between groups using the Chi-squared test. NRS-11 pain levels, which are non-interval ratings, were compared between groups using Mann–Whitney *U* test. The alpha-type error was set at 5 %. To assess the diagnostic value of the 11 symptoms (other than pain) between SAP and SIP, a multiple logistic regression model was fitted to the outcome variable SAP. All of the variables of the checklist were included in order to obtain a predicted probability for SAP for each individual patient. Because some teeth were bridge abutments and the question did thus not make sense, the variable “Tooth feels too high” had 11 missing values. These were multiply imputed with 5 replications. Results of the logistic regression are based on the pooled estimates of the 5 imputed data sets. The area under the receiver operating characteristic (AUC) curve was used to evaluate the discriminative ability of the regression model.

In an alternative approach recursive partitioning was used to construct a decision tree. The focus of the tree was to facilitate the diagnostic decision between SAP and SIP. All variables of the checklist were included in the tree model. All analyses were conducted using R statistical software [22].

## Results

### Study population and teeth

From January 2013 over a period of 15 month (64 weeks) 368 adult patients attended the dental emergency unit at our institution with severe pain on a permanent tooth. One hundred and forty-seven were not eligible to enter the study because they did not meet the criteria for inclusion (Fig. 1). From the 221 patients with acute pain of endodontic origin, 70 were diagnosed with SIP, 103 with SAP and 48 with AAA. The 48 individuals diagnosed with AAA were also excluded from analysis (Fig. 1). Of the remaining 173 patients diagnosed with either SIP or SAP, the ratio between females to males was 70/103. The average patient age was 40 years and ranged between 18 and 78 years. The ratio between mandibular and maxillary teeth was 107/66. One hundred and thirty of the teeth were molars, 33 premolars and 10 anterior teeth. One hundred and forty-seven of the teeth were multi-rooted, and the remaining 26 were single-rooted. Eleven of the 103 teeth diagnosed with SAP were root-filled. There were no statistical differences regarding tooth types between SIP and SAP in the current cohort (*P* > 0.05).

### Pain levels

Pain levels were statistically similar (*P* > 0.05) for both conditions under investigation. Median NRS-11 ratings were 8 for both SAP and SIP, with similar inter-quartile ranges: 2 for SAP, 1 for SIP. There was also no difference (*P* > 0.05) in pain levels between male and female patients. Eighty-one percent (81 %) of the patients used analgesics within the last 24 h before seeking emergency treatment. There was no statistical difference between the two conditions under investigation in this regard either.

### Key symptoms

**Fig. 2:**
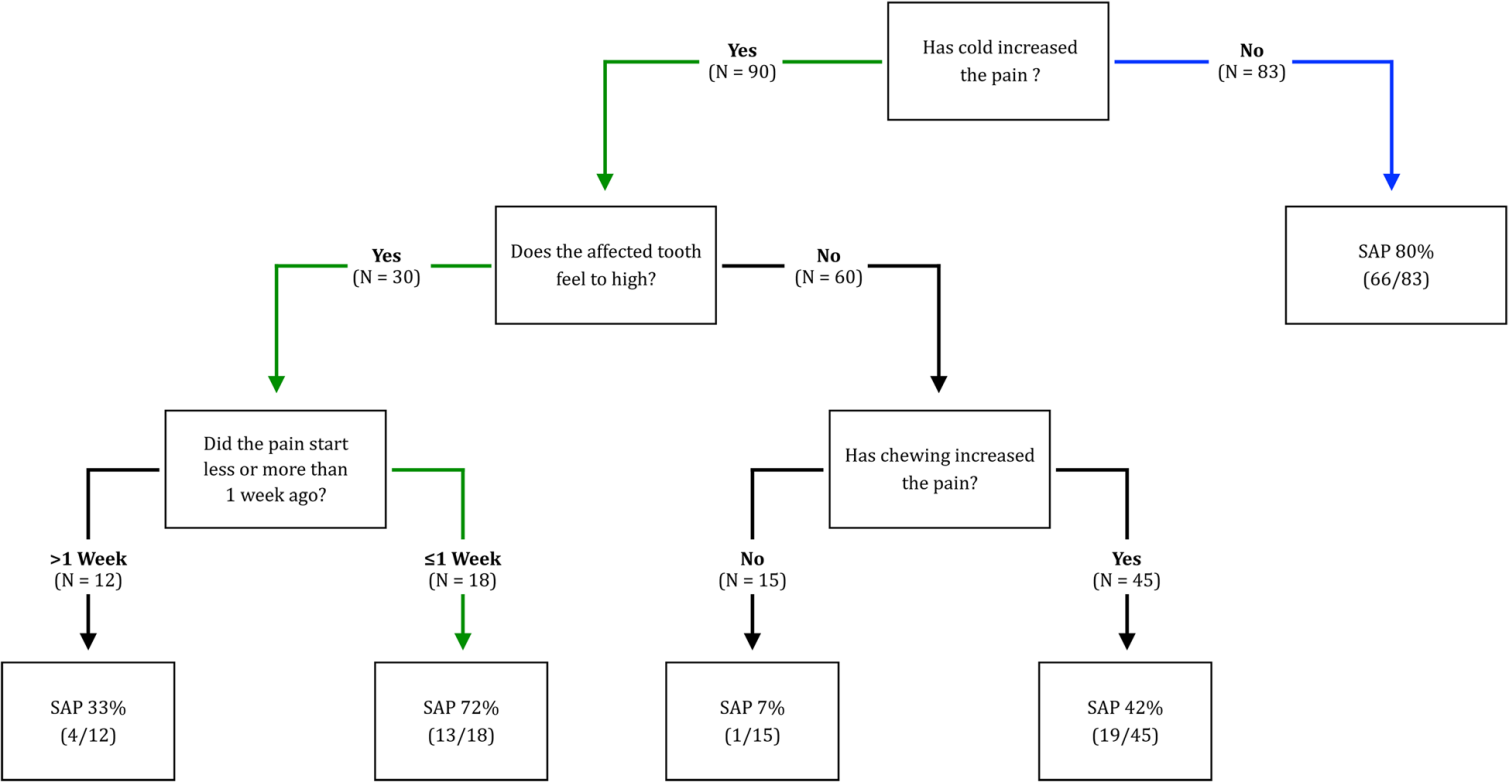
Decision tree to identify symptomatic apical periodontitis (SAP) based on recursive partitioning. SAP is the condition that can have systemic consequences. Green arrows indicate a set of symptoms that lead to a likely diagnosis of SAP in teeth with a history of pain to cold stimuli

Assessment of the checklist revealed that large differences in the symptomology of SAP and SIP were found for pain duration, pain on cold, and the feeling that the tooth was too high (Table 3). The prediction model, based on the multiple imputed data set, is summarized in Table 4. Furthermore, the estimated odds ratios and 95 % confidence intervals (CI) are displayed. The discriminative ability of the prediction model resulted in an AUC of 0.796 (95 % CI: 0.728–0.864). The decision tree analysis (Fig. 2) resulted in a tree with splits according to pain on cold, awareness of the tooth feeling too high, and pain duration. The first indicator for SAP was a reported absence of pain to cold stimuli. In teeth that did have a history of pain triggered by cold stimuli, the decision tree correctly identified SAP in 72 % of the teeth that felt too high and had hurt for less than one week. The overall sensitivity of the tree was 95 % and the specificity was 31 %. The positive predictive value was 67 %.

**Table 3:** Descriptive statistics: counts of symptoms in patients diagnosed with SIP (*N* = 70) and SAP (*N* = 103)

Question	SIP	%	SAP	%
Sleep disturbed	56	80.0 %	87	84.5 %
Pain has decreased	11	15.7 %	12	11.7 %
Pain less 1 week	32	45.7 %	72	69.9 %
Constant pain	25	35.7 %	51	49.5 %
Radiating pain	25	35.7 %	26	25.2 %
Sharp pain	38	54.3 %	37	35.9 %
Pain on chewing	49	70.0 %	89	86.4 %
Pain on hot	28	40.0 %	29	28.2 %
Pain on cold	53	75.7 %	37	35.9 %
Cold lessens pain	5	7.1 %	18	17.5 %
Tooth feels high^a^	20	28.6 %	50	48.5 %

*SIP* symptomatic irreversible pulpitis, *SAP* symptomatic apical periodontitis

^a^This variable had 11 missing values (teeth were bridge abutments)

**Table 4:** Results of the prediction model for SAP

	Odds ratio	95 % CI
Sleep disturbed	1.0	0.4–2.6
Pain has decreased	0.9	0.3–2.8
Pain less 1 week	2.1	1–4.4
Constant pain	1.6	0.8–3.4
Radiating pain	0.6	0.3–1.5
Sharp pain	0.5	0.2–1
Pain on chewing	2.5	1–6.3
Pain on hot	0.8	0.4–1.6
Pain on cold	0.2	0.1–0.5
Cold lessens pain	0.8	0.2–2.9
Tooth feels high	1.9	0.9–4.3

*SAP* symptomatic apical periodontitis, *CI* confidence Interval

## Discussion

The current study related symptoms to clinical findings. From an immediate treatment planning perspective, SAP is the more critical condition, and its diagnosis should not be missed [16]. It was confirmed that the reported presence or absence of pain to cold stimuli was a first differentiator between the clinical diagnoses of SAP and SIP. Moreover, decision analysis identified additional symptoms associated with a diagnosis of SAP also in teeth with a history of pain to cold stimuli. If these felt too high and had hurt for less than one week, the probability that SAP was diagnosed was still 72 %. A set of questions was thus identified that should be helpful in clinics and for cross-sectional studies to discern between SAP and SIP.

The current study is limited by the fact that data were generated in one single city. The relative frequency of the conditions under investigation is influenced by demographics, the local health care system and socio-economic factors [23]. It would appear that in older studies, SIP was more frequent than SAP [24], while newer investigations including the current work found the opposite [3]. This could be due to the fact that caries, the main cause for symptomatic pulpitis [9, 25], has steadily declined in industrialized countries [26]. Acute forms of apical periodontitis including abscess formation, on the other hand, can occur as late complications in crowned teeth and/or root-filled counterparts [27].

While current pulp tests and two-dimensional radiology are insufficient to determine the exact histological condition of asymptomatic teeth [28–30], the methods that were used in the present study to differentiate between the two acute conditions under investigation can be regarded as sound. The teeth diagnosed with SIP mostly showed a delayed, yet always more pronounced reaction to the cold test compared to healthy counterparts. It has been shown that painful teeth responding with an delayed, but increased and lingering response to the cold test invariably contain a vital pulp or at least vital aspects of the pulp in the apical root canal [25, 28]. In multi-rooted teeth some roots may still contain vital tissue that responds to thermal tests, while in other roots the tissue can be partially necrotic [31]. These vital aspects of the pulp inhibit bacterial infection [32]. Consequently, it is fair to state that acutely painful teeth with positive response to cold test differ from counterparts that test negatively in regard to the level of infection. An intra-operative diagnosis upon entering the pulp space was not performed to differentiate between SIP and SAP.

It is known that the intake of analgesics can affect endodontic diagnosis. The pain perception itself can decrease, or the response to clinical test like tooth percussion can be reduced [33]. However, the teeth under investigation were severely painful. Although 81 % (*N* = 140) of the 173 patients included to this study had consumed analgesics within the past 24 h before seeking emergency treatment, 79 % (*N* = 136) of them reported severe pain at levels between 7 and 10 on a NRS-11 scale [21]. Moreover, almost all teeth (92 %; *N* = 159) featured a painful response to percussion. In contrast the influence of analgesics on pulpal sensitivity test has shown to be negligible [34]. Consequently, the influence of analgesics on the diagnostic procedures performed here is expected to be low. It needs to be acknowledged that SIP and SAP are clinically defined separate diagnostic entities based on a set of criteria defined by international consensus (and as such reported in textbooks and major journal articles; [6]). SIP, SAP, and later AAA are biological, dynamically evolving stages of the same underlying bacterial infection. The transition from pulpitis or chronic apical periodontitis to acute apical periodontitis is clinically important, because it marks the point when a spread of the infection from the pulp space to the periapical tissues is about to occur [4]. However, the transitions between these conditions are rarely clear-cut [32]. Furthermore, transitions from one stage to the next can be fast. This is reflected in the current data in that 35.9 % of the patients diagnosed with acute SAP reported a history of sensitivity to cold (Table 3). Almost half of SAP teeth did not show clear apical radiolucencies typical for chronic apical periodontitis (Table 2). This can also be taken as an indicator that the inflammatory conditions in the periapical tissues developed more rapidly than any radiologically discernible bone changes occurred. This is in line with observations published by other authors [10]. Some recent research suggests that bone changes do occur early in the disease process, when the pulp is still vital [35]. These changes, however, are not necessarily detected on single-tooth radiographs [36]. Cone beam computed tomography (CBCT) was shown to be more sensitive in detecting apical disease [36]. The observation on the dynamics of periapical bone changes made here warrants further investigation and verification using CBCT.

Earlier authors concur with the present results in that pain to cold stimuli [28] and the feeling that the affected tooth is too high [13] can help to differentiate between SIP and SAP. However, it has to be cautioned that these authors did not use the current nomenclature of the diseases under investigation, and thus direct comparisons are limited. The current finding that sensitivity to cold is the main indicator for an inflamed vital pulp corresponds to the observations made in the only study on this topic in humans with induced pulpal inflammation [37]. Consequentially, this should further the support of cold testing e.g., carbon dioxide snow being the main clinical test, in conjunction with radiographic images, to diagnose SIP [25]. The patients diagnosed with SAP reported significantly more often the perception that the pain-causing tooth felt too high compared to patients diagnosed with SIP. This observation may be explained by the spread of the inflammation to the periapical ligament. The accumulation of inflammatory exudate may extrude the affected tooth, thus rendering it tender to occlusion [15]. Other common clinical tests were performed in this study, but these were of little value in differentiating between the acute forms of the conditions under investigation. As an example, 159 (92 %) of the 173 teeth in this study were positive to percussion, with no difference between the two conditions. This is in line with published reports: the percussion test has little to no diagnostic value [28, 38]. This is especially the case with painful teeth [39]. Nevertheless, the test is used in the current AAE nomenclature [6]. In accordance with the AAE terminology each tooth that is sensitive to percussion has a periapical diagnosis of “symptomatic apical periodontitis [10, 40]. In the current report, however, the comprehensive diagnosis of the acute forms of SAP before abscess formation versus SIP was based on the thermal responsiveness of the pulp (Table 2).

Numerical rating scales have been validated, and are commonly used for the assessment of pain intensities [7, 41]. The NRS-11 came to use here because of the interview character of this study. In contrast to a visual analogue scale, which is also commonly used in studies on endodontic pain, the NRS can be verbally applied without any visual aids. The 11 potentially differentiating symptoms used in this communication were selected based on the clinical experience of the investigators and common textbook recommendations [20]. They have been assessed, albeit not in the precise formulation attempted here, in various previous studies to differentiate between teeth of different clinical conditions [13, 28]. Pain intensity of endodontically involved teeth causing the patient to seek emergency care was investigated in several previous studies [2, 7, 40, 42]. The current mean NRS-11 pain levels between 7 and 8 correspond well to those measured using a VAS scale on emergency patients in previous studies [7, 42]. The current findings confirm an earlier report in that pain intensity had no differentiating value in the context of acute endodontic conditions [7]. This was not the case for pain *duration* though. Teeth affected by pulpits apparently hurt for more than one week before the pain reached a level that caused the patient to seek emergency care.

The current approach to facilitate the diagnostic decision between SAP and no SAP (SIP) has been two-fold. Initially, a prediction model including all available clinical information was constructed to assess the discriminative ability of the full checklist as measured with the AUC. The resulting AUC was nearly 80 %, which is an indication that the full model is in some degree valuable for the distinction between patients with SAP and SIP. However, the use of a prediction model based on 11 variables may be difficult to implement in a clinical setting unless a respective computer algorithm will be made available, e.g., in the form of a software application. For that reason, the decision tree analysis, which facilitates the distinction into the groups of SAP and SIP following a specific order of questions/key symptoms, was added in a second step (Fig. 2). The overall sensitivity to detect SAP based on the decision tree was 95 %. However, as is typical for diagnostic decisions with a high sensitivity, the resulting specificity of the tree was lower (31 %). This result demonstrates that the decision tree can be useful for cross-sectional studies. In a clinical setting, the decision tree may be helpful to advise patients during out-of hours calls.

## Conclusions

This study confirmed that in severely painful teeth, the most specific single symptom to differentiate between SIP and SAP was pain to cold stimuli. In addition, however, decision analysis identified a set of key symptoms to diagnose SAP also in teeth with a history of pain to cold stimuli. If these felt too high and had hurt for less than one week, the probability that SAP was diagnosed was still 72 %.

## Abbreviations

AAA, acute apical abscess; AAE, American Association of Endodontists; AUC, area under the receiver operating characteristic curve; CBCT, cone beam computed tomography; NRS, numeric rating scale; SAP, symptomatic apical periodontitis; SIP, symptomatic irreversible pulpitis
